# A Framework for Generating Radial and Surface-Oriented Regularized Stokeslets

**DOI:** 10.3390/fluids7110351

**Published:** 2022-11-14

**Authors:** Nicholas G. Chisholm, Sarah D. Olson

**Affiliations:** Department of Mathematical Sciences, Worcester Polytechnic Institute, Worcester, MA 01609, USA

**Keywords:** regularized stokeslets, regularization error, smoothing factor, boundary integral methods

## Abstract

Error in the method of regularized Stokeslets is highly dependent on the choice of the blob or regularization function that is utilized to handle singularities in the flow. In this work, we develop a general framework to choose regularizations at the level of the vector potential via smoothing factors. We detail the derivation for radial smoothing factors and specify properties which ensure that the solution is a regularized flow satisfying the incompressible Stokes equations. Error analysis is completed for both the far-field flow (away from the location of the forces) as well as at the location of the forces, relating our newly derived smoothing factors to commonly used blob functions and moment conditions. When forces are on a surface, we extend the radial smoothing factor case to the case of non-radial regularizations that are surface-oriented. We illustrate the utility of this framework by computing the forward and inverse problems of a translating sphere using radial and surface-oriented regularizations.

## 1. Introduction

There are many low Reynolds number biological flows at the microscale where viscous forces dominate. Such examples include flows due to swimming bacteria or sperm [1–4], those created within the cytoplasm of a living cell by molecular motors or microtubules [5–9], as well as those within microfluidic devices [10,11]. In this regime, when stresses are linearly related to strain, we assume that these flows are governed by the incompressible Stokes equations, given by

(1a) $$\mu \nabla^{2}u-\nabla p+f=0,$$

(1b) ∇⋅u=0,

where μ is the fluid viscosity, u is the fluid velocity, p is the pressure, and f is the external force density on the fluid. In this work, we restrict our focus to three-dimensional fluid flows and assume μ=1.

The fundamental solution to (1), known as a “Stokeslet”, represents the response of an unbounded, otherwise quiescent fluid, to a singular point force F exerted at position y with corresponding force density f=Fδ(x−y) [12,13]. Here, x represents a point within the fluid, and δ is the Dirac delta distribution. Since (1) is linear, general Stokes flows can be obtained by superposing Stokeslets distributed at different points throughout the fluid domain and/or at domain boundaries. Utilization of the Stokeslet has led to many pioneering studies on flagellar beating [14–16], as well as the development of slender body theory [15,17]. In the case of an immersed structure in a flow, the force density f in (1) may be a function of time and/or space; when point forces are concentrated on surfaces that are not smooth, or curves in $R^{3}$, the velocity is singular.

Many numerical approaches have been developed to approximate the flow due to structures immersed in a fluid governed by (1). Boundary integral methods, such as the boundary element method or boundary collocation methods, are particularly advantageous because they reduce computation of a flow in $R^{3}$ to the evaluation of surface integrals on the domain boundaries. One challenge, though, is that these integrals are singular, and some effort must be expended to evaluate them properly [12,13]. The immersed boundary (IB) method, though not limited to Stokes flows, was developed to handle immersed elastic structures that deform in response to the flow and/or other conditions [18]. In the IB method, regularized point forces on the (discretized) structure interact with a “background” fluid; a moving Lagrangian grid and a fixed Eulerian grid are employed for the structure and fluid, respectively. A major advantage of the IB method is that the Eulerian (fluid) grid can be regular, and re-meshing of the fluid domain is therefore unnecessary when the Lagrangian grid of the structure deforms.

The method of regularized Stokeslets (MRS) [19,20] draws from both the IB method and classic boundary integral methods. It was developed to handle structures immersed in a fluid with a Lagrangian grid, paralleling the IB method, but for applications governed by the Stokes equations [19,20]. Like other methods in the boundary-integral family, the MRS may be derived from the boundary integral form of the Stokes equations, where the Stokeslet appears as the kernel. The approach of the MRS is to replace the singular Stokeslet with a regularized approximation, so, similar to the IB method, forces exerted at boundary points are regularized and spread into the fluid. Like other boundary-integral methods, there is no need for a Eulerian grid for the fluid. The use of a regularized Stokeslet also regularizes the singular boundary integrals that appear in boundary integral methods, simplifying the treatment of these integrals.

There are several ways in which the Stokeslet may be regularized. For example, one may replace the singular force density f=Fδ with $f=F\phi_{\epsilon }$, where $\phi_{\epsilon }$ is a mollifier or “blob” function that spreads force over a small characteristic distance ϵ; a regularized Stokeslet is given by the resulting solution to (1) [19,20]. Another method involves directly multiplying the singular terms of the Stokeslet by appropriate smoothing factors [21,22]; flow properties such as incompressibility are not guaranteed with this approach, but corrections can be derived.

The MRS has gained popularity for modeling structures immersed in a Stokesian flow due to its relative ease of implementation. To date, the MRS has been effectively applied to a wide range of applications such as sperm motility [23–25], bacterial motility [26], and other cellular flows. Through the use of regularized image systems, the MRS can be adapted to different types of fluid domains, including triply and doubly periodic domains, or domains that are bounded by a plane-wall or a spherical boundary [27–32].

As with all numerical approaches, there are different types of errors that can accrue. In the MRS, error accrues from quadrature rules for approximating the boundary integral, force and structure discretizations, and time-stepping algorithms [19,30,33–35]. At the core of the MRS is the choice of the blob function $\phi_{\epsilon }$, the properties it satisfies, and the size of the parameter ϵ that controls the size of the region to which the point force is spread. As an example, a very popular blob function originally proposed by Cortez [19] is

(2) $$\phi_{\epsilon }(r)=\frac{15\epsilon^{4}}{8\pi {\left(\|r{\|}^{2}+\epsilon^{2}\right)}^{7/2}},$$

where r=x−y measures position relative to the location of forcing y. The blob function given by (2) is a radially symmetric function with infinite support that approximates the Dirac delta distribution when ϵ is small. Since the spreading parameter ϵ controls the region to which the force is spread, studies have been completed to understand the relation between the discretization of immersed structures and the parameter ϵ for the blob function $\phi_{\epsilon }$ in (2) [36]. Other studies have focused on derivations and properties of other blob functions, including infinitely supported $\phi_{\epsilon }\prime s$ with algebraic decay and exponential decay [34,37], as well as $\phi_{\epsilon }\prime s$ with compact support [28,34,37].

Accuracy of the MRS depends heavily on $\phi_{\epsilon }$, which induces regularization error in the computed velocity field with magnitude that is different close to (near-field) and far away from (far-field) the structure or location of forces. The far-field regularization error is generally $O\left(\epsilon^{n}\right)$, where n is the order of the first non-vanishing moment of $\phi_{\epsilon }$, excluding the zeroth moment [20]. If $\phi_{\epsilon }$ has three planes of symmetry, then all odd moments vanish, and the error is generally $O\left(\epsilon^{2}\right)$. In the radially symmetric case, Zhao et al. [37] show that the far-field error is dominated by a potential dipole whose strength is proportional to the second moment of $\phi_{\epsilon }$. A far-field correction canceling this potential dipole is derived, leading to regularized Stokeslets with far-field error that decays at a rate similar to $\phi_{\epsilon }$, and this rate may be faster than algebraic. On the other hand, near-field regularization error is O(ϵ) for a general blob function [20]. Corrections to this error have been derived via local analysis of flow near the boundary [21,22,34]; specifically, near-field error can be reduced to $O\left(\epsilon^{3}\right)$ with a correction to the blob function, e.g., that given by (2) [34].

Here, instead of generating regularized Stokeslets by choosing a blob function $\phi_{\epsilon }$, we develop regularizations by appropriately smoothing potentials obtained from a Helmholtz decomposition of the Stokeslet velocity field. Potentials derived via Helmholtz decomposition have often proved useful for decomposing Stokes flows for different purposes. For example, Tran-Cong and Blake [38] express solutions to the Stokes equations in terms of Papkovich–Neuber potentials, and this formulation has been utilized to construct formulas for Stokes flows in a half space or in periodic domains [39,40]. The potentials that we use are not Papkovich–Neuber potentials, but they do appear as intermediate quantities in the derivation of the Papkovich–Neuber solutions to the Stokes equations [38,41].

For radially symmetric regularizations, we utilize a direct smoothing factor approach, similar to Tlupova and Beale [22], but our formulation has the advantage that it automatically ensures that fluid incompressibility is maintained. Far-field regularization error is easily controlled; for smoothing factors that decay sufficiently quickly in the far field, we find that the resulting blob functions have a second moment that vanishes automatically, and explicit correction is not required as in Zhao et al. [37]. It is also straightforward to derive regularized Stokeslets that satisfy certain near-field correction conditions, which offer improved accuracy. Another advantage of our newly derived framework is that the corresponding blob functions need not be radially symmetric, and we consider vector potentials which generate regularized Stokeslets associated with an axisymmetric force distribution. The orientation of these Stokeslets can be chosen to spread force predominantly along the surface of a structure. We illustrate the utility of these approaches by demonstrating how error can be reduced for the problem of a translating, rigid sphere through different choices of smoothing factors. Both forward and inverse problems are considered, where in the former case the surface traction on the sphere is known, but the fluid velocity is not. In the inverse problem, the velocity of the structure is known, and we seek the forces acting on the boundary.

## 2. Methods

At the heart of the method of regularized Stokeslets is the appropriate choice of a regularization to ensure that flow velocities of structures are nonsingular and have a controllable error that we can quantify. We first start by reviewing the singular fundamental solution and the solution for an assumed regularized force. Our new approaches are then presented for regularizing the biharmonic function at the vector potential level for both radial and surface oriented (non-radial) regularizations.

### 2.1. The Stokeslet and Regularized Stokeslet

Let F be a point force at y with corresponding force density f(r)=Fδ(r) where r=x−y. Restricting ourselves to $r\in R^{3}$ where u(r)→0 and p(r)→0 as r→∞, the fundamental solution to (1) is called the Stokeslet and is given by

(3) $$u_{S}\left(r\right)=G\left(r\right)\cdot F=\frac{1}{8\pi }\left(\frac{I}{r}+\frac{rr}{r^{3}}\right)\cdot F,$$

(4) $$p_{S}\left(r\right)=P\left(r\right)\cdot F=\frac{r}{4\pi r^{3}}\cdot F,$$

where r=‖r‖, and we have a quiescent flow in the absence of a point force. Noting that F is arbitrary and that (1) is linear in F, the second-order velocity tensor field G and pressure vector field P satisfy

(5a) $$\nabla^{2}G-\nabla P+I\delta \;=0,$$

(5b) ∇⋅G=0,

where I is the identity tensor.

As can be seen in (3), the solution is singular at r=0, which is an issue that must be overcome by any numerical method based on Stokeslets. The method of regularized Stokeslets avoids this issue by utilizing a mollifier or blob function $\phi_{\epsilon }$. This function is generally taken to be a smooth and bounded function that approximates the Dirac delta distribution δ, satisfying

(6) $$\underset{\epsilon \to 0}{\text{lim}}\;\phi_{\epsilon }(r)=\delta (r)\;\text{and}\;\int_{R^{3}}\;\phi_{\epsilon }(r)\text{d}r=1.$$

We also require that $\phi_{\epsilon }$ satisfies the scaling property

(7) $$\phi_{\epsilon }(r)=\epsilon^{-3}\phi_{1}(r/\epsilon ),$$

where $\phi_{1}$ is the blob function with ϵ=1. A widely used $\phi_{\epsilon }$ is given in (2). Importantly, the parameter ϵ controls the region over which the singularity is regularized; physically, one may think of ϵ as the length scale over which the point force on the fluid at y is spread. Given a regularized force density $f(r)=F\phi_{\epsilon }(r)$ where we have a point force at y and r=x−y, the corresponding regularized Stokeslet satisfies

(8a) $$\nabla^{2}G_{\epsilon }-\nabla P_{\epsilon }+\phi_{\epsilon }I=0,$$

(8b) $$\nabla \cdot G_{\epsilon }=0.$$

Again, solving (8) for $G_{\epsilon }$ and obtaining the regularized flow as $u_{\epsilon }=G_{\epsilon }\cdot F$ gives an exact solution for a given regularization of the forces.

A general Stokes flow u(x) can be approximated using regularized Stokeslets via the boundary integral formulation of (1) [13,20,42]. Assume we have a solid structure immersed in a fluid that does not change volume and that there are no (non-conservative) external body forces acting on the fluid. Let ∂D denote the set of points on the fluid-solid boundary. We can approximate the velocity at a point x as

(9) $$u\left(x\right)\approx u_{\epsilon }\left(x\right)={∭}_{y\in R^{3}}\;u\left(x\right)\phi_{\epsilon }\left(x-y\right)\text{d}V=\iint_{y\in \partial D}\;G_{\epsilon }\left(x-y\right)\cdot q\left(y\right)\text{d}S,$$

where the integral over the boundary ∂D is referred to as the single-layer potential, and q is a surface force density. If the solid structure moves as a rigid body, q(y) is the negative of the surface traction vector on the surface. The regularization error is the error incurred by approximating u by its convolution with $\phi_{\epsilon }$, denoted $u_{\epsilon }$. Recall that, for general $\phi_{\epsilon }$, this error can be shown to be $O\left(\epsilon^{n}\right)$ at points far from ∂D, where n is the order of the first nonvanishing moment of $\phi_{\epsilon }$, and O(ϵ) at points near ∂D [20]. We introduce the boundary integral equation given in (9) because we will exploit the idea of smoothing factors for integrand kernels to explore regularizations with specified properties.

### 2.2. A Regularization Approach Utilizing the Vector Potential of the Stokeslet

To solve for the regularized Stokeslet $G_{\epsilon }$, the solution to (8a,b), we first take the divergence of (8a). Then, using (8b), a Poisson equation for $P_{\epsilon }$ is obtained,

$$\nabla^{2}P_{\epsilon }=\nabla \phi_{\epsilon }.$$

Applying the Laplace operator $\nabla^{2}$ to both sides of (8a) and using the previous result shows that $G_{\epsilon }$ satisfies

(10) $$\nabla^{4}G_{\epsilon }=\left(\nabla \nabla -I\nabla^{2}\right)\phi_{\epsilon }.$$

Similar to Cortez [19], we assume that our blob function $\phi_{\epsilon }$ (not necessarily radial) can be written in terms of a scalar function $B_{\epsilon }$ that satisfies

(11) $$\nabla^{4}B_{\epsilon }=-\phi_{\epsilon }.$$

Substituting (11) into (10) and solving for $G_{\epsilon }$ gives

$$G_{\epsilon }=\left(I\nabla^{2}-\nabla \nabla \right)B_{\epsilon }+B_{H},$$

where $B_{H}$ is an arbitrary second-order-tensor satisfying $\nabla^{4}B_{H}(r)=0$ for all $x\in R^{3}$. However, since we require $G_{\epsilon }$ to vanish as r→∞ (where r=‖x−y‖), $B_{H}$ must also vanish, since regular solutions to $\nabla^{4}B_{H}=0$ are either constant or diverge as r→∞. Therefore,

(12) $$G_{\epsilon }=\left(I\nabla^{2}-\nabla \nabla \right)B_{\epsilon },$$

and we can use the identity $\left(\nabla \nabla -I\nabla^{2}\right)B=\nabla \times \nabla \times (IB)$, to rewrite (12) as

(13) $$G_{\epsilon }=\nabla \times A_{\epsilon }$$

where $A_{\epsilon }$ is given by

(14) $$A_{\epsilon }=-\nabla \times \left(IB_{\epsilon }\right)=-\varepsilon \cdot \nabla B_{\epsilon }$$

and ε is the permutation tensor. Equation (13) follows from the Helmholtz decomposition of the divergence-free vector field $G_{\epsilon }\cdot F$ for some force F. Thus, we refer to $A_{\epsilon }$ as the “vector potential” of $G_{\epsilon }$. We highlight that neither of the “potentials” $B_{\epsilon }$ nor $A_{\epsilon }$ are unique for a given regularization of the Stokeslet $G_{\epsilon }$. For example, an arbitrary curl-free vector field may be added to $A_{\epsilon }$ without changing the form of $G_{\epsilon }$.

We now consider a particular solution to (11) in the limit ϵ→0, i.e., with $\phi_{\epsilon }=-\delta$, given by B(r)=r/8π. This solution can be used to derive the singular Stokeslet using (12) with $\phi_{\epsilon }=\delta$ [13]. Using (14), the vector potential A derived from B is given by

(15) $$A(r)=-\varepsilon \cdot \nabla B=-\frac{\varepsilon \cdot r}{8\pi r}.$$

Though A(r) is bounded, it is undefined at the origin because ∇B=r/8πr fails to exist there, leading to singular terms in the corresponding velocity field in (3).

This observation suggests that generating a regularized Stokeslet can be accomplished by first regularizing the vector potential $A_{\epsilon }(r)$ and then applying (13). We regularized A by replacing ∇B in (15) with a smoothed approximation $\nabla B_{\epsilon }$ that is defined everywhere and satisfies ${\text{lim}}_{r\to \infty }\;\left[\nabla B_{\epsilon }(r)-\nabla B(r)\right]=0$. We do not strictly require ${\text{lim}}_{r\to \infty }\;\left[B_{\epsilon }(x)-B(x)\right]=0$ because we may add any constant to $B_{\epsilon }$ without affecting the corresponding form of $G_{\epsilon }$. Once $B_{\epsilon }$ is selected, we may generate a corresponding regularized Stokeslet according to (13) and (14).

An advantage of this approach is that the resulting regularized Stokeslet will automatically be divergence free, which is not necessarily true if the singular factors of (3) are considered separately. Moreover, we need not restrict ourselves to radial regularizations, and we may therefore consider surface-oriented distributions. One may also solve (8) using a particular choice of $\phi_{\epsilon }$, but closed-form solutions may not be available, especially if one considers non-radial choices of $\phi_{\epsilon }$.

### 2.3. Radially Symmetric Regularizations

#### 2.3.1. Formulation of Smoothing Factors

Radial regularizations, those which are associated with a radially symmetric blob function, are by far the simplest and most common type used in the MRS. If we assume that $B_{\epsilon }$ is radially symmetric, we find from applying the chain rule to (14) that

(16) $$A_{\epsilon }(r)=-\frac{\varepsilon \cdot r}{r}\frac{\;\text{d}B_{\epsilon }(r)}{\text{d}r}=8\pi A(r)\frac{\text{d}B_{\epsilon }(r)}{\text{d}r},$$

where, in the last equality, we have recognized the appearance of the singular vector potential A given in (15). This observation suggests letting $s_{\epsilon }(r)=8\pi \;\text{d}B_{\epsilon }/\text{d}r$, where $s_{\epsilon }$ is a “smoothing factor” that suitably regularizes A and only depends on r=‖r‖. Using the smoothing factor, we can rewrite $A_{\epsilon }$ as

(17) $$A_{\epsilon }\left(r\right)=-\frac{\varepsilon \cdot r}{8\pi r}s_{\epsilon }\left(r\right).$$

Substituting (17) into (13) gives the regularized Stokeslset in terms of $s_{\epsilon }$ as

(18) $$8\pi G_{\epsilon }\left(r\right)=h_{1}\left(\epsilon ;r\right)I+h_{3}\left(\epsilon ;r\right)rr,$$

where

$$h_{1}(\epsilon ;r)=\frac{1}{r}\frac{\;\text{d}\left(rs_{\epsilon }\right)}{\text{d}r}\;\text{and}\;h_{3}(\epsilon ;r)=-\frac{1}{r}\frac{\;\text{d}\left(r^{-1}s_{\epsilon }\right)}{\text{d}r}\text{.}$$

The subscripts are indicative of the rates at which $h_{1}$ and $h_{3}$ decay as r→∞, which are $r^{-1}$ and $r^{-3}$, respectively. Using (11), $\phi_{\epsilon }$ can be written in terms of $s_{\epsilon }$ as

(19) $$8\pi \phi_{\epsilon }\left(r\right)=-\nabla^{2}\nabla \cdot \left(\frac{r}{r}s_{\epsilon }\left(r\right)\right)=\frac{{\text{d}}^{3}s_{\epsilon }}{\text{d}r^{3}}-\frac{4}{r}\frac{{\;\text{d}}^{2}s_{\epsilon }}{\text{d}r^{2}}.$$

There are properties that the smoothing factor $s_{\epsilon }$ must satisfy so that, when used in (18), it produces a regularized Stokeslet that is suitable for use with the MRS. A suitable regularization is one in which $G_{\epsilon }$ is bounded everywhere (including at r=0), approximates G for r≫ϵ, and which has an associated blob function $\phi_{\epsilon }$ that satisfies (6). The necessary properties are stated by the following theorem:

**Theorem 1.** *Let the smoothing factor* $s_{\epsilon }\in {𝒞}^{3+k}$ *for* k>0 *be a function that satisfies the following properties:*

(20a) $$s_{\epsilon }(r)=s_{1}(r/\epsilon ),$$

(20b) $$s_{1}\left(r\right)=s_{1}^{\prime \prime }\left(r\right)=O\left(r^{m}\right)\;\text{for}\;m\geq 1\;\text{as}\;r\to 0,$$

(20c) $$\underset{r\to \infty }{\text{lim}}\;s_{1}\left(r\right)=1,$$

*where the primes indicate differentiation. Then,* $s_{\epsilon }$ *gives a regularized Stokeslet that is suitable for the MRS by satisfying the following criteria:*

1. *The regularized velocity field scales as $G_{\epsilon }(r)=\epsilon^{-1}G_{1}(r/\epsilon )$ and blob function scales according to* (7).
2. *The regularized velocity field* $G_{\epsilon }$ *is bounded and the blob function* $\phi_{\epsilon }$ *is also bounded.*
3. $\;\phi_{\epsilon }$ *integrates to unity over* $R^{3}$, *as required by* (6).

**Proof.** Equation (20a) is a scaling property which guarantees that the blob function $\phi_{\epsilon }$ obeys the related scaling property (7), as can be seen by substituting (20a) into (19). The similar scaling property of $G_{\epsilon }$ follows from substituting (20a) into (18). That s and $s^{\prime \prime }$ vanish at a linear rate as r→0, as required by (20b), ensuring that $G_{\epsilon }$ and $\phi_{\epsilon }$ are bounded at the origin, which can be shown by examining (18) and (19) in the limit of r→0. In addition, $G_{\epsilon }$ and $\phi_{\epsilon }$ are bounded and continuous for r>0 since we have assumed that $s_{\epsilon }$ is bounded and has continuous derivatives up to the third order. Proof that $\phi_{\epsilon }$ integrates to unity is contained within Appendix A, where we relate the moments of $\phi_{\epsilon }$ to $s_{\epsilon }$. In particular, from (A4), we find that $\phi_{\epsilon }$ integrates to unity as long as the properties of Theorem 1 are satisfied. □

#### 2.3.2. Error Analysis

At this point, there are a large number of smoothing functions $s_{\epsilon }$ that could be utilized that satisfy the three properties given in (20). We would like to choose $s_{\epsilon }$ such that the effect of the regularization error is as small as possible. We define the regularization error of the Stokeslet velocity field as $E_{\epsilon }=G-G_{\epsilon }$, which is found from (18) to be

(21) $$E_{\epsilon }\left(r\right)=\left[1-s_{\epsilon }\left(r\right)\right]G\left(r\right)+\frac{s_{\epsilon }^{\prime }\left(r\right)}{8\pi }\left(I-\frac{rr}{r^{2}}\right),$$

where, as we recall, G is the *singular* Stokeslet.

The far-field regularization error is the error at points where r≫ϵ, far from the point of forcing. From (18), we find that the magnitude of $E_{\epsilon }$ for fixed r≫ϵ simply mirrors the rate at which $1-s_{\epsilon }$ vanishes for fixed r as ϵ→0. Due to (20a), $1-s_{\epsilon }$ and $s_{\epsilon }^{\prime }$ vanish at similar rates; if $1-s_{\epsilon }(r)=1-s_{1}(r/\epsilon )=O\left(\epsilon^{n}\right)$ for fixed r≫ϵ, then $s_{\epsilon }^{\prime }(r)=\epsilon^{-1}s_{1}^{\prime }(r/\epsilon )=O\left(\epsilon^{n}\right)$ too, since $s_{1}^{\prime }(r/\epsilon )=O\left(\epsilon^{n+1}\right)$. A similar fact holds if $1-s_{\epsilon }(r)$ vanishes at an exponential rate, in which case $E_{\epsilon }$ represents an exponentially small correction for r≫ϵ. In principle, one could also define a smoothing factor that is exactly unity beyond a distance ϵ, in which case $G_{\epsilon }=G$ for r≫ϵ and the far-field error vanishes exactly. In this case, the corresponding blob function will have compact support on a ball of size ϵ according to (19). Others have dealt with compactly supported blobs [28,34,37], and we restrict ourselves to blobs with infinite support.

A detailed far-field error analysis by Zhao et al. [37] of the flows produced by general blob functions $\phi_{\epsilon }$ shows that a potential dipole generally dominates the error for r≫ϵ; this error is $O\left(\epsilon^{2}\right)$ regardless of how quickly $\phi_{\epsilon }$ decays. To achieve better accuracy, an explicit correction is needed to eliminate the potential dipole contribution, with the corrected blob having a vanishing second moment. In our framework, an explicit far-field correction is unnecessary; as shown in (A4) in Appendix A, the second moment of $\phi_{\epsilon }$ vanishes identically whenever $1-s_{\epsilon }(r)$ vanishes at a sufficient rate. In particular, we find from (A2) that, if

(22) $$1-s_{\epsilon }(r)=O\left(\epsilon^{2+\alpha }\right)$$

where α>0, then contributions to $E_{\epsilon }$ that are $O\left(\epsilon^{2}\right)$ or larger are filtered out.

Equation (21) describes the error for a single regularized Stokeslet at some point in the fluid, but we would also like to know the error in the global velocity field u(x) produced by the regularized boundary integral equation given in (9). This equation produces an exact result when ϵ→0 and $G_{\epsilon }$ is essentially replaced by G. Since the singular Stokeslet is given by “adding back” the error term in (21) to the regularized Stokeslet, $G=G_{\epsilon }+E_{\epsilon }$, we find that the error in the velocity field associated with a known q is

(23) $$e_{\epsilon }\left(x\right)=u\left(x\right)-u_{\epsilon }\left(x\right)=\iint_{y\in \partial D}\;E_{\epsilon }\left(x-y\right)\cdot q\left(y\right)\text{d}S.$$

At points near the boundary, near-field regularization error dominates $e_{\epsilon }$ and is known to generally be O(ϵ) [20]. However, (21) does not yield useful information about the near-field error due to the singular nature of $G_{\epsilon }$. Thus, it is necessary to perform a local analysis of the velocity error given in (23) at points near the boundary, as is detailed in several previous works [21,22,34,43–45]. We keep our discussion of this correction brief and restrict our attention to $e_{\epsilon }$ for points exactly on the boundary, since we will find this helpful in accurately computing the drag on a rigid sphere. Since the kernel $E_{\epsilon }(r)$ of (23) is highly localized, one may expand the integrand in (23) using a local set of coordinates for a chosen point $y=y_{0}\in \partial D$ centered on a small patch on the boundary. Integration over a radial set of coordinates (ϱ,ϑ) in the pre-image space, where $y_{0}={\left.y\right|}_{\varrho =0}$, yields the leading terms of the velocity error as

(24) $$e_{\epsilon }=\;\frac{\epsilon }{8\pi }\int_{0}^{2\pi }\;\;\int_{0}^{\infty }\;\;\left[1-s_{1}(\varrho )\right](I+\overset{ˆ}{\varrho }\overset{ˆ}{\varrho })\cdot q\left(y_{0}\right)\text{d}\varrho \;\text{d}\vartheta \;+\frac{\epsilon }{8\pi }\int_{0}^{2\pi }\;\;\int_{0}^{\infty }\;\;\varrho s_{1}^{\prime }\left(\varrho \right)\left(I-\overset{ˆ}{\varrho }\overset{ˆ}{\varrho }\right)\cdot q\left(y_{0}\right)\text{d}\varrho \;\text{d}\vartheta +O\left(\epsilon^{\text{min}\;\left(3,\beta \right)}\right),$$

where we assume $1-s_{\epsilon }(r)$ is $O\left(\epsilon^{\beta }\right)$ for β>0 as ϵ→0. All of the angular dependence of the integrands on ϑ in (24) is contained in the radial unit vector $\overset{ˆ}{\varrho }$. Factoring the nested integrals, we see that the O(ϵ) contributions to $e_{\epsilon }$ vanish to $O\left(\epsilon^{3}\right)$ at $y=y_{0}$ if $s_{\epsilon }$ satisfies the additional property

(25) $$\int_{0}^{\infty }\;\left[1-s_{\epsilon }(\varrho )\right]\text{d}\varrho =\int_{0}^{\infty }\;\varrho s_{\epsilon }^{\prime }(\varrho )\text{d}\varrho =0.$$

Note that the two integrals above are actually equivalent; one can show this fact by integrating the second integral by parts and using the properties of the smoothing factor (20). The near error field analysis of Nguyen and Cortez [34] produced a similar correction condition that $\int_{0}^{\infty }\;\varrho^{3}\phi_{\epsilon }(\varrho )\text{d}\varrho =0$. Indeed, this condition can be shown to be equivalent to (25) by using (A3). Note that (25) is purely a property of the smoothing factor and does not depend on the geometry of the boundary.

We now have a framework to generate several different smoothing factors $s_{\epsilon }$ that result in regularized fluid flows with known error properties. In Section 3.1, we demonstrate how this framework leads to several useful regularized Stokeslets that have already appeared in the literature and also formulate several new ones that perform well in accurately resolving the drag on a translating sphere. We now develop a related framework for regularizations that have symmetry about an axis but are not radially symmetric.

### 2.4. Surface-Oriented Regularizations

The method of regularized Stokeslets most commonly employs radial regularizations, which distribute force evenly in all directions at each discretization point. Regularized Stokeslets associated with non-radial distributions of force density (blob functions) have been employed much less frequently. Cortez [46] considered the use of “Stokeslet segments”, non-radially regularized Stokeslets derived by continuously distributing a radially regularized Stokeslet along a line segment with a linearly varying force. In the case of a rigidly translating slender rod, Stokeslet segments are shown to produce lower error in the no-slip boundary condition placed on the centerline of the rod as well as improved agreement in the computed drag with slender body theory. Tyrrell et al. [47] considered “Stokeslet rings” for use with axisymmetric bodies, which, similar to Stokeslet segments, distribute regularized forces along a circle. Stokeslet segments and Stokeslet rings are advantageous because the forces are in some sense distributed more evenly along the boundary of the structure (and hence the fluid domain). Compared to the standard method employing radial regularizations, these modified distributions of forces on segments and rings reduce the discretization error of (9), where the “exact” surface force density q(y) typically varies continuously along points y on the boundary of the structure ∂D.

Here, we introduce a different modification to the method of regularized Stokeslets guided by similar intuition but based on using non-radial regularized Stokeslets that are oriented along the surface normal of the structure boundary in the fluid at each discretization point. We see from (9) that, for a given point on the fluid boundary, force is spread locally over a plane normal to the boundary rather than isotropically. This observation suggests using a regularization that spreads force more widely along this plane (along the boundary) than normal to it (away from the boundary). Therefore, we consider an axisymmetric (but non-radially symmetric) regularization about a line which passes through a point on the surface and is parallel to the surface normal at that point. Thus, the regularized Stokeslet has an orientation that varies from point to point and coincides with the orientation of the boundary.

In Section 2.3, we illustrated how we could generate regularized Stokeslets with radial $\phi_{\epsilon }$ by multiplying the singular vector potential given by (14) by a smoothing factor. Unfortunately, we cannot apply exactly the same approach in the non-radial case; while the vector potential still exists according to (14), it is more difficult to regularize directly without violating (8a) by inducing a forcing term that is not of the form $\phi_{\epsilon }I$. Recall that the smoothing factor arose in (17) as $s_{\epsilon }=\text{d}B_{\epsilon }/\text{d}r$, where $B_{\epsilon }$ is assumed to be radially symmetric. Our approach in this case will be to develop an approximation $B_{\epsilon }$ to B, where $B_{\epsilon }$ is not necessarily radially symmetric, such that the associated regularized flow is bounded and approximates the singular Stokeslet as ϵ→0. This will require that $B_{\epsilon }$ be sufficiently smooth. We also require that the associated blob function is an approximate Dirac delta distribution. To this end, we employ the following theorem.

**Theorem 2.** *Suppose that the biharmonic potential* $B_{\epsilon }$ *obeys the scaling property*

(26) $$B_{\epsilon }(r)=\epsilon B_{\epsilon }(r/\epsilon )$$

*and satisfies*

(27) $$\underset{\epsilon \to 0}{\text{lim}}\;\left(I\nabla^{2}-\nabla \nabla \right)\left[B_{\epsilon }-B\right]=\underset{\epsilon \to 0}{\text{lim}}\;G_{\epsilon }-G=0,$$

*where the first equality follows from* (12) *and* B(r)=r/8π. *Additionally, suppose that* $B_{\epsilon }\in {𝒞}^{k}$ *for* k≥5. *Then*, $G_{\epsilon }$, *as given from* $B_{\epsilon }$ *by* (12), *is a bounded*, ${𝒞}^{k-2}$ *approximation to the singular Stokeslet, and* $\phi_{\epsilon }$, *given by* (11), *satisfies the properties expected of a blob function, given by* (6) *and* (7).

**Proof.** Plugging (26) into (11) renders a blob function that satisfies the scaling property given by (7). Then, we can show that $\phi_{\epsilon }$ satisfies (6) as follows. Taking the trace of both sides of (10) yields

(28) $$\phi_{\epsilon }=-\frac{1}{2}\nabla^{2}\;\text{tr}{\;G}_{\epsilon }.$$

Since $\phi_{\epsilon }$ is continuously differentiable, we can integrate (28) over a volume $V_{R}$ that contains all points within a sphere of radius r=R from the origin and then apply the divergence theorem to the right-hand side to give

(29) $$\int_{r\in V_{R}}\;\phi_{\epsilon }\left(r\right)\text{d}V=-\frac{1}{2}\int_{r\in \partial V_{R}}\;\left(\overset{ˆ}{n}\cdot \nabla \right)\;\text{tr}{\;G}_{\epsilon }\left(r\right)\text{d}S,$$

where $\overset{ˆ}{n}$ is the outward facing unit normal vector of $\partial V_{R}$. Taking the limit as ϵ→0 of (29) and using (6), $\phi_{\epsilon }$ is replaced by δ and $G_{\epsilon }$ with G in (29), giving the identity

(30) $$\int_{r\in V_{R}}\;\delta \left(r\right)\text{d}V=-\frac{1}{2}\int_{r\in \partial V_{R}}\;\left(\overset{ˆ}{n}\cdot \nabla \right)\;\text{tr}\;G(r)\text{d}A=1.$$

Now, for small but finite ϵ, we may consider (29) in the limit R→∞ of (29). Since the difference between $G_{\epsilon }(r)$ and G(r) becomes arbitrarily small as r/ϵ→∞, we may replace $G_{\epsilon }$ on the right-hand side of (29) by G to within an arbitrarily small error. Then, comparing (30) and (29) and taking the integrals to be over $R^{3}$ shows that $\phi_{\epsilon }$ satisfies (6). □

While there are a large number of potential choices for $B_{\epsilon }$ that will produce suitably regularized flows associated with non-radial blob functions, we focus on one example that possesses a single axis of symmetry, assumed here to be the z-axis, and distributes more force along the plane normal to the axis than along the axis. We start by letting

$$B^{a}(r)=\frac{1}{16\pi }\left(\sqrt{z^{2}+(\rho -a{)}^{2}}+\sqrt{z^{2}+(\rho +a{)}^{2}}\right)$$

where $r=\rho \overset{ˆ}{\rho }+z\overset{ˆ}{z}$ with $\rho^{2}=x^{2}+y^{2}$ and $\overset{ˆ}{\rho }$ and $\overset{ˆ}{z}$ denoting the unit vectors in the ρ and z directions. $B^{a}$ is symmetric about the z-axis, has isosurfaces that are oblate spheroids with a “focal circle” of radius d lying in the xy-plane, and satisfies (27). On the focal circle at z=0 and $\rho =\pm d,\nabla B^{a}$ fails to exist, but this is similar to ∇B (which has spherical isosurfaces) failing to exist at the origin for a singular Stokeslet. Since we want the Stokeslet velocity field and force profile $\phi_{\epsilon }$ to be bounded at all points, we regularize $B^{a}$ as

(31) $$B_{\epsilon }^{a}(r)=\frac{1}{16\pi }\left(\sqrt{z^{2}+(\rho -a\epsilon {)}^{2}+\epsilon^{2}}+\sqrt{z^{2}+(\rho +a\epsilon {)}^{2}+\epsilon^{2}}\right)$$

where ϵ is the regularization parameter and a is an O(1) constant. The biharmonic potential given in (31) approaches the singular biharmonic potential B(r)=r/8π as ϵ→0 up to an $O\left(\epsilon^{2}\right)$ regularization error for r≫ϵ. Note that (31) reproduces the 7/2-power-law blob given by (2) if a=0. Without loss of generality, we hereafter let a=1/2. An explicit formula for $G_{\epsilon }^{a}$ is given in Appendix B.

### 2.5. Test Case: A Translating Sphere

To test the accuracy of the regularized Stokeslets we have thus far constructed, we apply them to the problem of a sphere of radius R=1 translating at unit velocity in an otherwise unbounded, quiescent fluid. A no-slip condition is imposed on the sphere as ${\left.u\right|}_{r=1}=𝒰$, where ‖𝒰‖=1. Since the sphere translates as a rigid body, −q is equal to the surface traction on the sphere surface. The analytical solution is well known to be q=3μ𝒰/2R, which simplifies to q=3𝒰/2 since we assume viscosity, velocity, and sphere radius are all unity. The drag D on the sphere is found by integrating q (a constant in this case) over the sphere surface, giving the classic Stokes drag result of $D=4\pi R^{2}\|q\|=6\pi \mu R\|𝒰\|=6\pi$.

We employ a Fibonacci lattice to generate a discrete set of n points on the sphere that has approximately equal spacing and is therefore convenient to use as a set of numerical quadrature points [48]. The k-th point is given by the formula

(32) $$\left(\varphi_{k},\theta_{k}\right)=\left[\frac{2\pi k}{\tau },{\text{cos}}^{-1}\;\left(1-\frac{2k-1}{n}\right)\right],$$

where the sphere is parameterized by polar angle φ and azimuthal angle θ.

As is standard in the method of regularized Stokeslets, (9) is approximated by letting $u_{\epsilon }=u$, inducing a regularization error, and the integral on the right-hand side is replaced with a discrete sum, inducing a discretization error. Thus, we have

(33) $$u\left(x\right)=\sum_{k=1}^{n}\;G_{\epsilon }\left(x-y_{k}\right)\cdot F_{k},$$

where $F_{k}=w_{k}q\left(y_{k}\right)$, and $w_{k}$ is the quadrature weight for point k. Note that $F_{k}$ has units of force and represents the discrete force exerted on the fluid by a regularized Stokeslet placed at point k. In the case of the surface-oriented Stokeslet described in Section 2.4, (33) is slightly modified as

(34) $$u\left(x\right)=\sum_{k=1}^{n}\;G_{\epsilon }^{a}\left(\overset{ˆ}{n}\left(y_{k}\right),x-y_{k}\right)\cdot F_{k},$$

where $G_{\epsilon }^{a}(\overset{ˆ}{n},r)=\left(I\nabla^{2}-\nabla \nabla \right)B_{\epsilon }^{a}(\overset{ˆ}{n},r)$ depends on the outwards unit normal vector $\overset{ˆ}{n}$ to the sphere at point $y_{k}$. Recall that (31) expresses $B_{\epsilon }^{a}$ in terms of ρ and z, where we assumed that $\overset{ˆ}{n}$ was aligned with the z-axis. Here, the dependence of $B_{\epsilon }^{a}$ on $\overset{ˆ}{n}$ is made explicit by letting $z=\overset{ˆ}{n}\cdot r$ and $\rho =\|(I-\overset{ˆ}{n}\overset{ˆ}{n})\cdot r\|$ in (31).

We address both the forward and inverse problem. In the forward problem, we assume that the quadrature weights are uniform, $w_{k}=w=4\pi /n$. In this case, the quadrature error associated with the Fibonacci grid defined by (32) is $\sim n^{-2}$ [49]. The velocity field may be computed for any point x by directly using (33) with $q_{k}=q=3/2$. In the inverse (Dirichlet) problem, we instead assume that the fluid velocity is known, $u\left(y_{j}\right)=U$, while the forces $F_{k}$ are unknown. The inverse problem is solved by evaluating (33) at $x=y_{j}$ to form a linear system of n equations given by

(35) $$u\left(y_{j}\right)=\sum_{k=1}^{n}\;G_{\epsilon }\left(y_{j}-y_{k}\right)\cdot F_{k,}$$

which are solved for the forces $F_{k}$. When surface-oriented Stokeslets are used, $G_{\epsilon }\left(y_{j}-y_{k}\right)$ in (35) is replaced with $G_{\epsilon }^{a}\left(\overset{ˆ}{n}\left(y_{k}\right),y_{j}-y_{k}\right)$.

**Figure A1.**
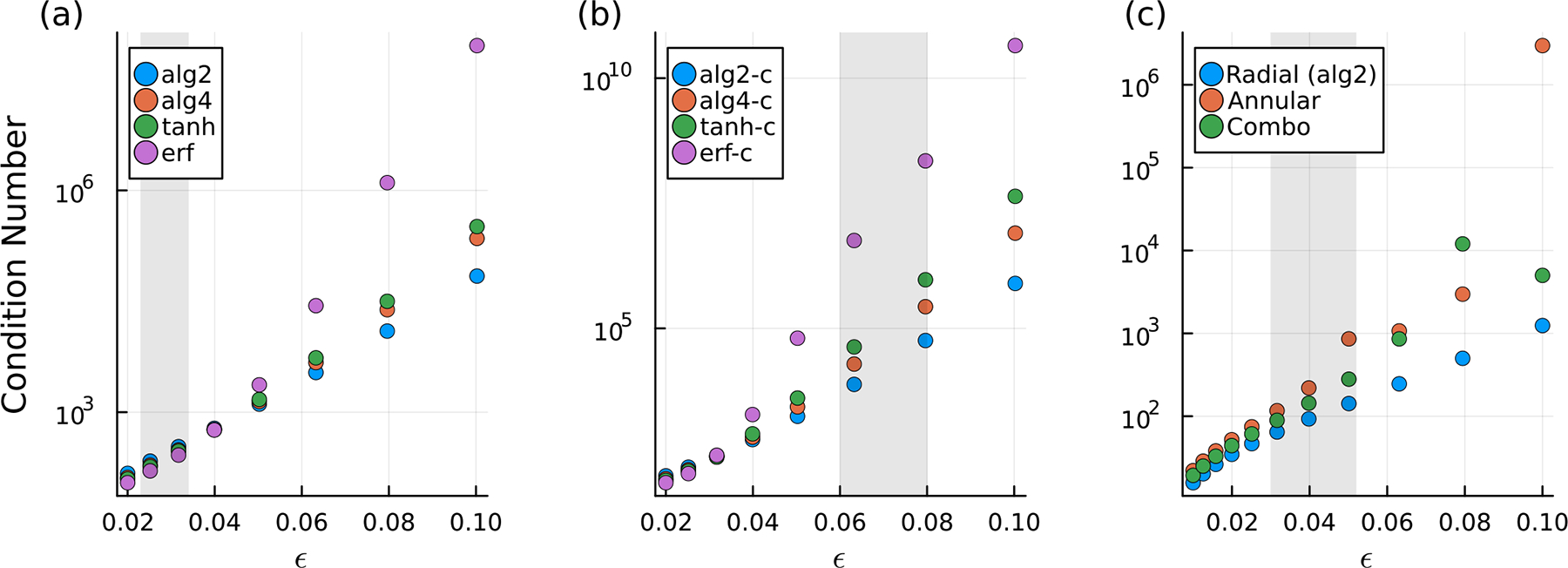
Skeel’s condition number for the inverse problem of a translating sphere described in Section 2.5 using (**a**) uncorrected radial, (**b**) corrected radial, and (**c**) surface-oriented regularizations. For the radial (**a**,**b**) and surface-oriented (**c**) regularizations, n=4096 and n=1024 discretization points are used, respectively. The shaded regions indicate the values of ϵ where the error in the drag is approximately minimized.

The linear systems produced by (35) are symmetric. They were solved using the Bunch–Kaufman [50] factorization routine (also called block ${LDL}^{T}$) provided by the LinearAlgebra module of the Julia v1.8 standard library, which internally calls the LAPACK routine dsytrf. Condition numbers of the matrices resulting from (35) are reported in Appendix C, Figure A1. Note that explicit formation of the matrix is only required in the inverse problem.

## 3. Results

### 3.1. Radial Regularizations

#### 3.1.1. Example Smoothing Factors

There are many choices of the smoothing factor $s_{\epsilon }$ that satisfy the three conditions given by (20) of Theorem 1. Recalling that $s_{\epsilon }(r)=s_{1}(r/\epsilon )$, Table 1 summarizes four smoothing factors $s_{1}$ hereafter employed as examples. These smoothing factors were chosen due to their simplicity and for their differing behavior for r≫ϵ.

Smoothing factors labeled “alg2” and “alg4” approach unity at an algebraic rate; the numerals 2 and 4 correspond to the power p where $1-s_{\epsilon }^{\text{alg}p}$ is $O\left(\epsilon^{p}\right)$ for fixed r≫ϵ. Coincidentally, we find that $s_{\epsilon }^{\text{alg2}}$ leads to the the commonly employed 7/2 power-law blob given in (2). We also find that $s_{\epsilon }^{\text{alg}4}$ leads to an algebraic blob function previously derived by demanding that the blob have a vanishing second moment and therefore a far-field regularization error of $O\left(\epsilon^{4}\right)$ [37]. In our framework, recall that the second-order moment of $\phi_{\epsilon }^{\text{alg4}}$ vanishes identically because $1-s_{\epsilon }^{\text{alg}4}=O\left(\epsilon^{2+\alpha }\right)$ where α=2>0. We note that, in the case of the alg2 regularization, the second moment of $\phi_{\epsilon }^{\text{alg}2}$ is nonvanishing and equal to 3/8π. In this case, one finds that (A4) with $s_{1}=s_{1}^{\text{alg2}}$ leading to a divergent integral, and thus (A4) cannot be used to directly evaluate the second moment of $\phi_{\epsilon }^{\text{alg2}}$. The regularizations labeled “tanh” and “erf” are named for smoothing factors equal to the hyperbolic tangent function and error function, respectively. The erf smoothing factor is related to the exponential blob derived by [51], and the tanh smoothing factor appears to lead to a regularization that is novel. We emphasize that many additional smoothing functions $s_{\epsilon }$ (and corresponding blob functions $\phi_{\epsilon }$) can be readily derived from this framework. In practice, evaluation of the regularized Stokeslet requires formulas for the functions $h_{1}$ and $h_{3}$, which appear in (18) and are derived from the smoothing factor of a particular regularization. Formulas for these functions are given in Appendix B, Table A1.

The corrections given in Table 1 are additional terms added to the original smoothing factors to satisfy the boundary velocity correction condition in (25). For example,

$$s_{1}^{\text{alg}2\text{-c}}(r)=\frac{r}{\sqrt{r^{2}+1}}+\frac{r}{{\left(r^{2}+1\right)}^{3/2}}$$

is the algebraic decay smoothing factor alg2 where we append a “-c” to the label to indicate a smoothing factor including the boundary velocity correction. These corrections must be chosen such that the original smoothing factor plus correction continues to satisfy the conditions of Theorem 1. For example, the correction terms must vanish at the origin and approach unity for r→∞. The corresponding blob functions, given by (19), are also shown in Table 1. To our knowledge, these blob functions have not appeared in previous literature.

Unless otherwise noted, we hereafter normalize the regularizations given in Table 1 by making the substitution $r\to \sqrt[{3}]{\phi_{1}(0)}r$ in the arguments to $s_{1}$. The normalized regularizations have blob functions that satisfy $\phi_{1}(0)=1$ and continue to satisfy (6) due to the scaling property (7). The former property is convenient because it makes the force distribution represented by each of the blobs easier to compare. Note that $\phi_{1}(0)$ is generally O(1) before normalization, ranging from about 0.4 to 6 for all regularizations considered.

**Figure 1.**
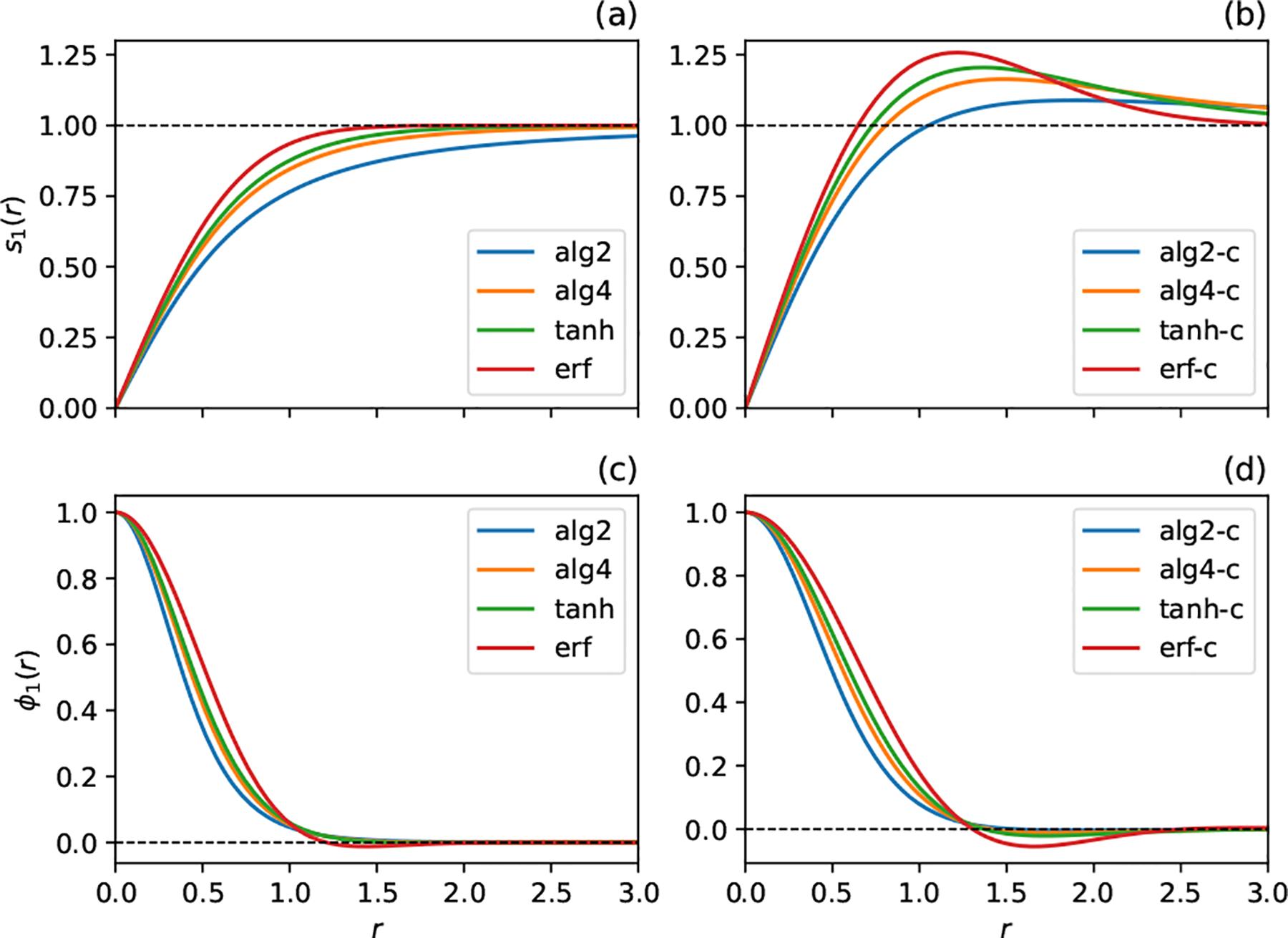
Behavior of the radially symmetric smoothing factors $s_{1}$ (**a**,**b**) and blob functions $\phi_{1}$ (**c**,**d**) given in Table 1, plotted as a function of r=‖r‖. All smoothing factors have been normalized by substituting $r\to r\sqrt[{3}]{\phi_{1}(0)}$ in the arguments to $s_{1}$, and the blob functions correspond to the normalized smoothing factors. No boundary corrections are utilized in (**a**,**c**) whereas (**b**,**d**) incorporates the boundary-velocity correction given by (25).

Plots of the normalized smoothing factors and blob functions specified in Table 1 are shown in Figure 1. All of the uncorrected smoothing factors increase monotonically to unity as r→∞, while the corrected smoothing factors increase above one and reach a maximum before approaching their limiting value in the far field. As is characteristic of blob functions that integrate to unity, $\phi_{1}\prime s$ with a higher maximum at r=0 decay faster. Additionally, most of the blob functions (all except alg2) are not monotonically decreasing but rather become negative and then increase, vanishing as r→∞. This feature is characteristic of blob functions which have a vanishing second moment, or, equivalently, are derived from a smoothing factor that vanishes faster than $O\left(\epsilon^{2}\right)$ as ϵ→0.

#### 3.1.2. Forward Problem

We now apply the radial regularizations described in Section 2.3 to the problem of a translating sphere described in Section 2.5. Unless otherwise noted, calculations in this section were performed using n=4096 discretization points. We also need to choose a value for ϵ. Given that the surface area of the unit sphere is 4π and that the discretization points are distributed close to evenly, setting $\epsilon ≲\epsilon_{0}=2n^{-1/2}\approx 0.03$ will prevent significant overlap of blobs at neighboring points, while $\epsilon ≳\epsilon_{0}$ will induce strong interaction between neighboring blobs. Thus, we may use $\epsilon_{0}$ as a reference value when considering different choices for ϵ.

**Figure 2.**
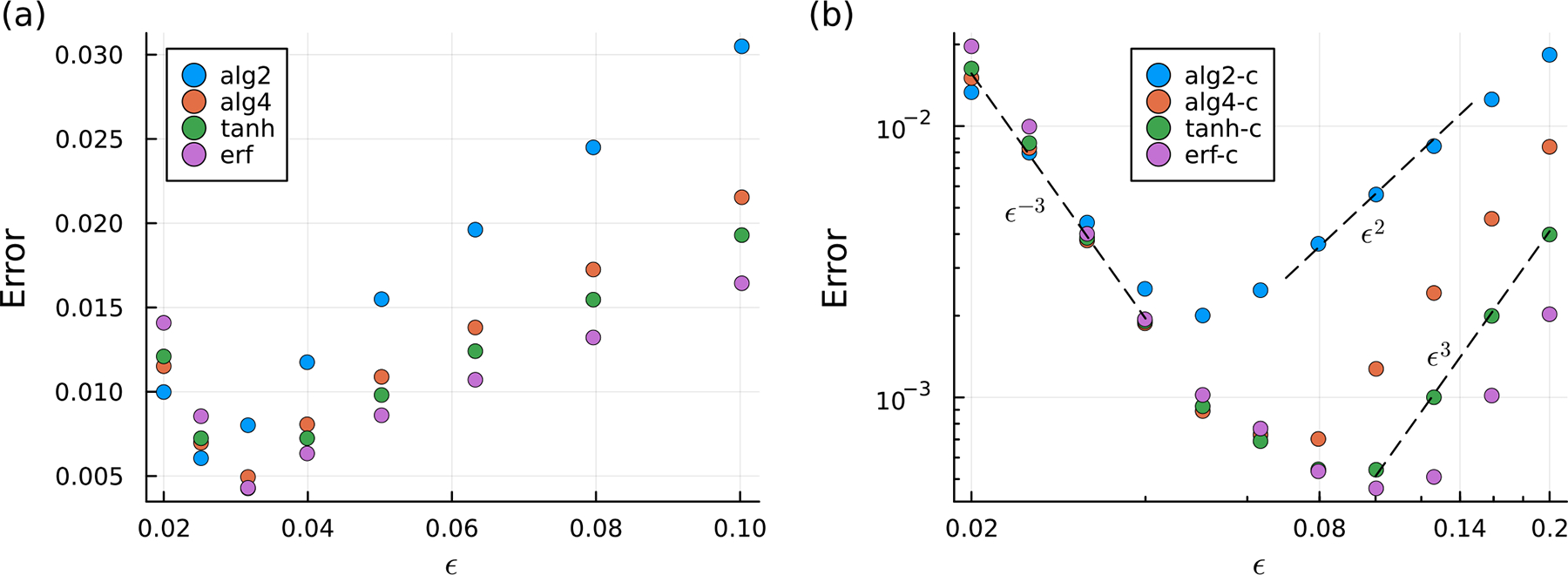
Error (sup-norm) in the computed velocity field on the sphere boundary versus the regularization parameter ϵ for the forward problem of a unit sphere translating at unit velocity 𝒰 in a fluid with unit viscosity. Here, the surface traction is prescribed as the constant vector q=3𝒰/2. The error is given as the sup-norm of u(x)−𝒰 for x in the set of discretization points on the sphere surface. The regularizations listed in the legend are those derived from the radial smoothing factors given by Table 1. Smoothing factors utilized in (**a**) leave the boundary velocity uncorrected whereas in (**b**), corrections according to (25) are included. In (**b**), a log-log scale is used to show the power-law dependence (dashed lines) of the error on ϵ in the discretization-error- and regularization-error-dominated regimes.

First, we consider the forward problem and compute the fluid velocity at each of the discretization points $y_{k}$ using (33) given the known surface traction of $q_{k}=3/2$ at each point. We then evaluate the error e(x)=u(x)−𝒰 and report the sup-norm (maximum) of ‖e‖ as ϵ is varied from 0.02 to 0.1 (Figure 2). The error can be considered as a sum of the regularization error and the quadrature error. The regularization error $e_{\epsilon }$ depends on ϵ as given by (23). The quadrature error is also known to depend on the regularization parameter for a fixed number of quadrature points as $\epsilon^{-3}$, and increases as ϵ is made small, in contrast to the regularization error, which decreases with ϵ [20]. Thus, we expect that the regularization error is small and the quadrature error dominates for smaller values of ϵ, whereas the regularization error dominates for larger values of ϵ. As a result, MRS calculations often exhibit a value of $\epsilon =\epsilon_{*}$ where the error is minimized for a given problem [20], which is apparent in Figure 2. The precise point at which this minimum occurs depends on the regularization used.

Regularizations derived from uncorrected smoothing factors—those that do not satisfy (25)—exhibit error minima at $\epsilon =\epsilon_{*}\approx \epsilon_{0}=0.03$ (Figure 2a), whereas corrected regularizations exhibit minima $\epsilon_{*}>\epsilon_{0}$ (Figure 2b). The near-field regularization error is reduced in the corrected case, shifting the transition from quadrature- to regularization-dominated error—and hence $\epsilon_{*}$—to larger values of ϵ and reducing the overall error at all but the smallest values of ϵ. We also observe approximate power-law dependence of $e_{\epsilon }$ on ϵ when regularization error is dominant $\left(\epsilon >\epsilon_{*}\right)$, as predicted by (23). For uncorrected regularizations, we find an O(ϵ) scaling in this regime, while, for corrected regularizations, we find an $O\left(\epsilon^{3}\right)$ dependence, except in the case of the alg2-c regularization where it is $O\left(\epsilon^{2}\right)$. This latter scaling is due to the fact that $1-s_{\epsilon }^{\text{alg2-c}}=O\left(\epsilon^{2}\right)$, and thus β=2 in (23). On the other hand, β=4 for the alg4-c regularization and, for the exponentially decaying erf and tanh regularizations, β is effectively infinite. Finally, we find that $e\sim \epsilon^{-3}$ in the quadrature-error-dominated regime $\left(\epsilon \ll \epsilon_{*}\right)$, which is an error scaling predicted previously by Cortez et al. [20].

#### 3.1.3. Inverse Problem

**Figure 3.**
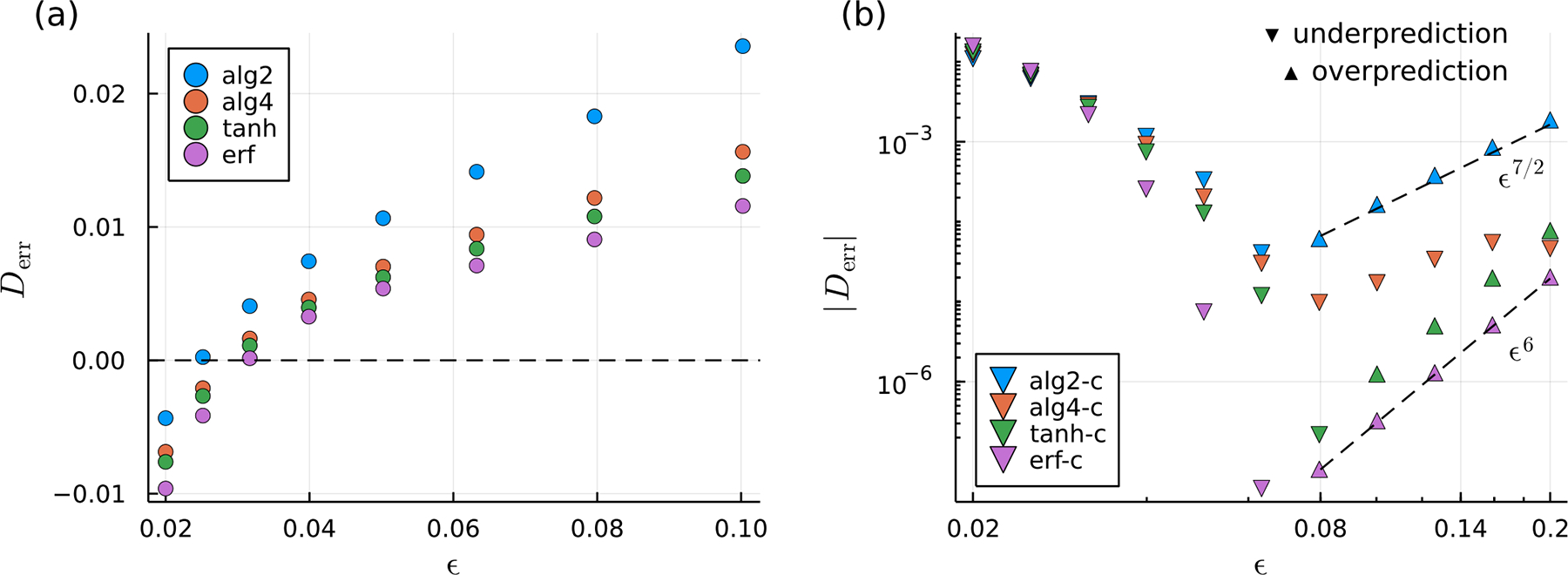
Similar to Figure 2, but for the corresponding inverse problem. Here, the error is the relative error with respect to the analytical result for the drag on the sphere, $D_{\text{err}}=D/6\pi -1$, where D is the drag. Results for uncorrected and corrected smoothing factors are shown in (**a**) and (**b**), respectively. In (**b**), a log-log scale is used, and the absolute value of $D_{\text{err}}$ is plotted. We use ‘▼’s to indicate under-prediction $\left(D_{\text{err}}<0\right)$ and ‘▲’s to indicate drag over-prediction $\left(D_{\text{err}}>0\right)$. The dashed lines indicate the power-law scaling of $D_{\text{err}}$ in the regularization-error-dominated regime.

We now consider the corresponding inverse problem and compute the drag on a sphere assuming rigid body translation at unit velocity, as described in Section 2.5. Recalling that the analytical result for the drag is 6π, we define the error in the numerically computed drag as $D_{\text{err}}=D/6\pi -1$. Figure 3a shows the error in the drag for the four uncorrected regularizations, while Figure 3b shows the error for the corrected regularizations. We find that the drag is generally under-predicted for small ϵ and over-predicted for large ϵ. Over-prediction in the case where ϵ is large can be rationalized by imagining that the blobs produce a sphere of effective radius $R_{\text{eff}}>1$ and recalling that the drag D=6πμR‖𝒰‖ increases with the radius. For smaller values of ϵ, under-prediction of the drag is due to insufficient overlap of the blob functions; a significant portion of the surface of the sphere exerts no force on the fluid, and the sphere therefore becomes “leaky”. While ${\left.u\right|}_{r=1}=𝒰$ is enforced exactly at the discretization points, fluid is allowed to penetrate the sphere boundary at points in between the dicretization points. We note that these error trends further motivate the potential use of surface-oriented regularizations described in Section 2.4. The rate at which the minimum is approached as ϵ is made smaller is dependent on the smoothing factor.

Like in the forward problem, quadrature error dominates for small ϵ and regularization error dominates for large ϵ. Hence, we similarly observe error minima at some particular value of $\epsilon =\epsilon_{*}$ for each regularization (Figure 3). The value of $\epsilon_{*}$ and the rate at which the error increases with ϵ for $\epsilon >\epsilon_{*}$ depends on the specific regularization used. Note, however, that $\epsilon_{*}$ and the scaling behavior of the error with ϵ in the inverse problem generally differ from those found for the forward problem. The uncorrected regularizations give error minima of $\epsilon_{*}\approx \epsilon_{0}=0.03$, and for $\epsilon >\epsilon_{0}$, the error depends linearly with ϵ, as it did in the forward problem (Figure 3a). The results for the corrected regularizations are more interesting. In Figure 3b, we find that, except at the smallest values of ϵ, the drag error is significantly smaller than that of the uncorrected smoothing factors. The error quickly decreases with ϵ until a minimum is reached and then increases at rates that approximately obey power laws, with the alg2-c regularization following a $\epsilon^{7/2}$ trend and the tanh-c and erf-c Stokeslets following an $\epsilon^{6}$ trend. In the regularization-error-dominated regime, the drag is over-predicted, while it is under-predicted for smaller ϵ, similar to the trends observed for the uncorrected regularizations. The alg4-c regularization is unique in that it only ever under-predicts the drag for the entire range of ϵ considered. It also does not exhibit a clear power law scaling. Overall, the erf-c regularization exhibits the smallest error followed by the tanh-c regularization, both generally producing smaller error than the algebraic regularizations (alg2-c and alg4-c). This observation suggests that there is a benefit to using smoothing factors whose far-field regularization error vanishes very rapidly, especially for inverse problems.

**Figure 4.**
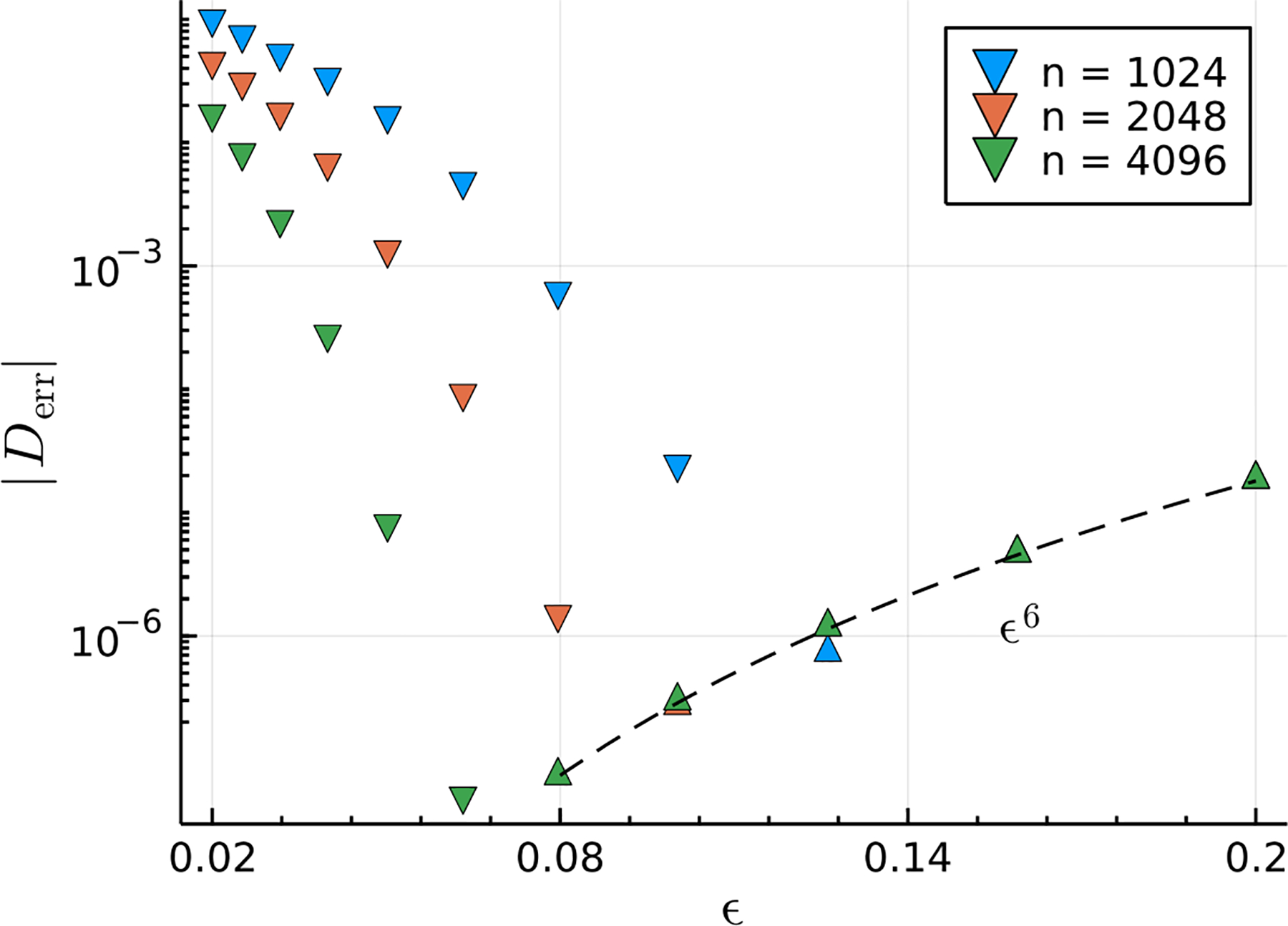
Error in the computed drag versus number of discretization points n using the erf-c regularization. The absolute value of the error is shown, but the direction of the triangle indicates the sign, following the same convention as that in Figure 3.

The effect of varying the number of discretization points n is shown in Figure 4 for the erf-c regularization. As more discretization points are used, the numerical quadrature represented by the summation in (35) becomes more accurate, and we expect the error to decrease. This reduction in error occurs mostly as expected, and the value of ϵ where the error is minimized shifts to smaller ϵ as the number of discretization points increases. Interestingly, past the respective error minimum for each value of n, the error collapses onto a common line that follows the same $\epsilon^{6}$ scaling as is shown in Figure 3. These observations indicate that regularization error is strongly dominant over quadrature error in the power-law scaling regime, where the error is apparently independent of the number of discretization points used. Using more discretization points does nothing to improve accuracy beyond the value of ϵ where the error minimum occurs.

### 3.2. A Surface-Oriented Regularization

**Figure 5.**
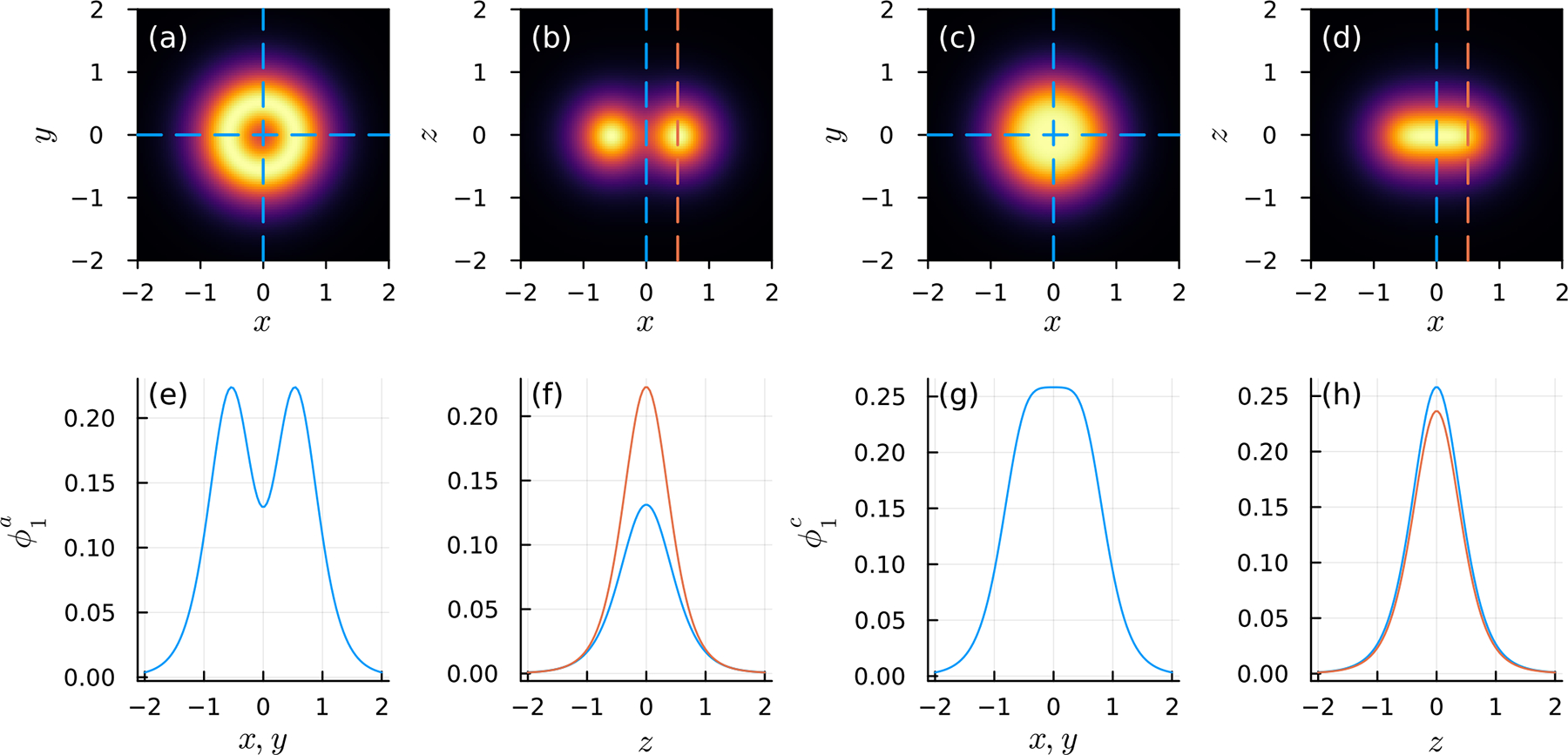
Force density profiles of the surface-oriented regularized Stokeslets, derived from (31), with ϵ=1. The orientation is along the z-axis. (**a,b**) plot the cross section of $\phi_{1}^{a}$ through the xy-and xz-planes, respectively, and (**c,d**) similarly plot $\phi_{1}^{c}$. A detailed profile of $\phi_{1}^{a}$ along the x- (or y-) axis is shown in (**e**), as well as parallel to the z- axis for ρ=0 and ρ=1 in (**f**). Similar plots of $\phi_{1}^{c}$ are shown in (**g**,**h**). Values sampled along particular lines in (**e–h**) are indicated by dashed lines in (**a–d**), where there is a correspondence between columns of figures. For example, the blue and orange dashed lines in (**b**) respectively correspond to the blue and orange force density profiles in (**f**).

Cross sections of the blob function $\phi_{\epsilon }^{a}$ associated with $B_{\epsilon }^{a}$ (with ϵ set to unity), given by (31), are plotted in Figure 5a,b,e,f. There, we find that the force density on the fluid near the point of forcing has an annular profile with the majority of the force concentrated near a circle of radius ϵ/2. The annular shape is not completely unexpected; we know from (31) that the gradients of $B_{\epsilon }^{a}$ are nearly singular on the circle ρ=ϵ/2 and z=0 for ϵ≪1. Force is distributed more widely on the xy-plane than along the z-axis, as desired. One might guess that the corresponding flow is that due to regularized Stokeslets distributed evenly along a ring. However, this flow is known to involve elliptic integrals [47], while that derived from $B_{\epsilon }^{a}$ has an algebraic form.

The regularization does not evenly spread force along the plane. For example, $\phi_{\epsilon }$ is not maximized at the origin as was true of the radial regularizations described in Section 2.3. In terms of the numerical method, $\phi_{\epsilon }^{a}$, though not uniform, still leads to well behaved regularized flows. In the case that one desires a more even spread to the force density, we can “fill in” the region of lower force at the origin by using a linear combination of $B_{\epsilon }^{a}$ with the biharmonic potential $B_{\epsilon }^{r}$ of a radially regularized Stokeslet centered at the origin. Thus, we let $B_{\epsilon }^{r}=B_{\epsilon }^{\text{alg2}}=\sqrt{r^{2}+\epsilon^{2}}/8\pi$, which corresponds to the “alg2” entry of Table 1, and combines with $B_{\epsilon }^{a}$ as

(36) $$B_{\epsilon }^{c}=c_{r}B_{\epsilon }^{r}+c_{a}B_{\epsilon }^{a}.$$

The corresponding blob function is $\phi_{\epsilon }^{c}=c_{r}\phi_{\epsilon }^{r}+c_{a}\phi_{\epsilon }^{a}$, and therefore we require $c_{r}+c_{a}=1$ so that $\phi_{\epsilon }^{c}$ integrates to unity. The empirical choice of $c_{r}=3/11$ and $c_{a}=8/11$ produces a monotone force density $\phi_{\epsilon }^{c}$ that has a global maximum at the origin and distributes force nonradially but in a uniform manner (see Figure 5c,d,g,h).

**Figure 6.**
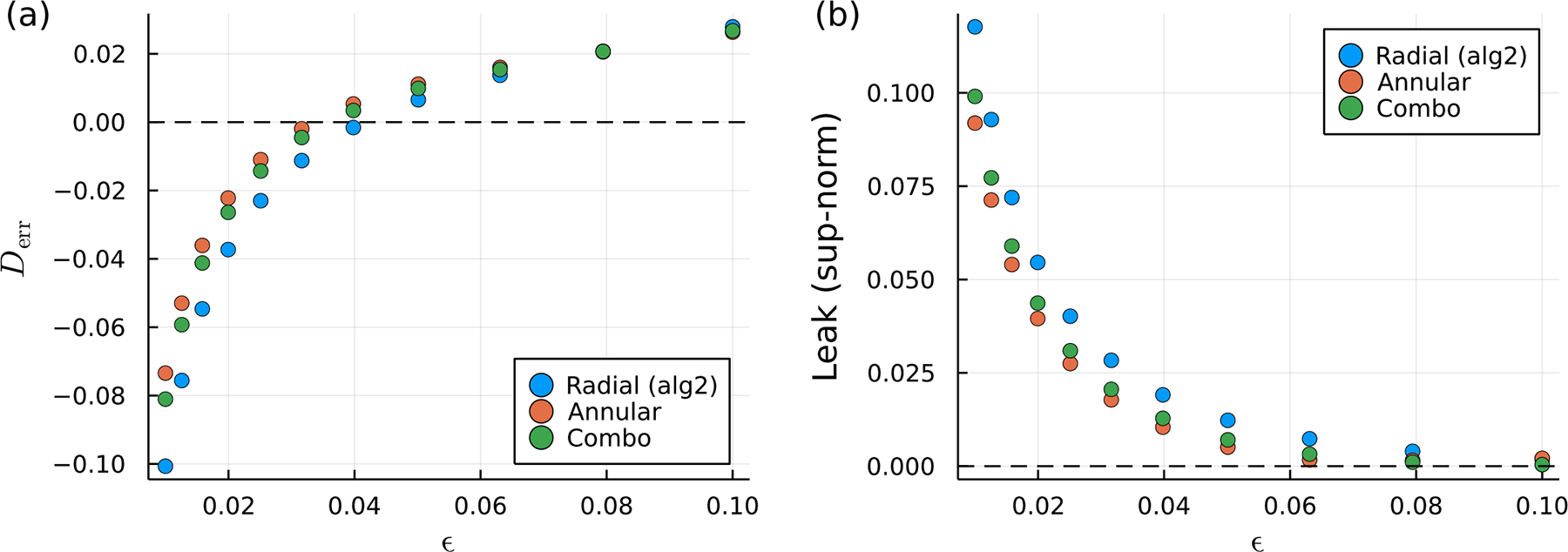
Error of surface oriented regularizations versus the radial alg2 regularization. The error in the drag is shown in (**a**) and the error in the boundary condition or “leak” at points on the surface of the sphere but in between the discretization points is shown in (**b**).

The drag on a translating sphere is resolved as an inverse problem, as described in Section 2.5, and compared to the radial alg2 regularization using n= 1024 discretization points. That is, we prescribe the velocity $u\left(y_{k}\right)=𝒰$ at the discretization points $y_{k}$ and solve (34) for the forces $F_{k}$ exerted at each point. The results, shown in Figure 6a, indicate reduced drag error for the surface-oriented regularizations compared to the radial regularization for the calculations performed for smaller values of ϵ. Here, the drag is under-predicted, but less so for the surface-oriented regularizations. For larger values of ϵ, the drag is over-predicted by all three of the regularizations. However, this over-prediction is not significantly worse for the surface-oriented regularizations than that of the radial regularization. Interestingly, the drag error is generally lower for the annular regularization than for the annular+radial regularization, which, as we recall, produces a more even distribution of force along the surface. This observation is a consequence of the linearity of the Stokes equations; linearly combining two regularizations in a “weighted average” results in a drag that is the same weighted average of the drag values.

We also examine the error in the no-slip boundary condition on the sphere surface. This error effectively vanishes at the discretization points because it is at these points where the boundary condition ${\left.u\right|}_{r=1}=𝒰$ is prescribed. However, away from the discretization points, this boundary condition is only satisfied approximately, and we refer to this kind of error as the “leak”, defined as ${\left.u\right|}_{r=1}-𝒰$. Therefore, we define another set of points on the sphere $y_{k}^{\prime }$ that are in between the discretization points by using (32), except where the $\theta_{k}$ are offset by an angle of π. The fluid velocities at these points are computed by reusing the forces $F_{k}$ that have already been determined from solving the inverse problem (using the original set of discretization points) and solving the corresponding forward problem by evaluating (34) for each $x=y_{k}^{\prime }$. The sup-norm of the leak measured at these points is shown in Figure 6b. We again find a reduced error for the surface-oriented regularizations, which have a smaller leak than the radial regularization. Intuitively, this follows from the fact that more of the force is spread along the boundary than normal to it, allowing less fluid to leak into the sphere. The annular regularization produces the lowest error, with the annular+radial regularization falling in the middle according to the weighted-average argument.

## 4. Discussion

We have developed a convenient framework for generating both radial and non-radial regularized Stokeslets for use with the MRS, which are automatically divergence-free and have easily controlled regularization error. Thus, we generate several examples expected to have different error properties. As a test case, we solved the forward and inverse problems of a steadily translating sphere using a selection of regularized Stokeslets generated from radially symmetric smoothing factors. We also solve the inverse problem using a regularized Stokeslet that distributes force in a surface-oriented manner. In general, we found the error in the fluid velocity at the sphere boundary (in the forward problem) and the drag (in the inverse problem) to depend heavily on the regularization used. For radial regularizations, the smoothing factors incorporating near-field error corrections for points on the boundary given by (25) proved valuable in reducing the overall regularization error, often by orders of magnitude.

For the forward problem of a translating sphere, the error in the velocity at the sphere boundary depends on ϵ in a manner consistent with previous error analyses of the MRS [20–22,34]. These error analyses, as well as our own discussion of error in Section 2.3.2, assume that the surface force density (or single-layer potential) q is the known input and that the velocity field is the output. For the inverse problem, the roles of input and output are reversed. Still, by using smoothing factors that correct for the “input” error in the velocity at the discretization points, the computed drag is much more accurate. Indeed, our results verify a claim by Nguyen and Cortez [34] that blobs including near-field correction terms will perform significantly better in inverse problems. However, there is no reason to expect that the error dependence on ϵ in the inverse problem is the same as in the forward problem, and our results reflect this fact. While the O(ϵ) error of the drag calculations performed using the uncorrected smoothing factors is the same as the expected O(ϵ) error in the velocity, the $D_{\text{err}}\sim O\left(\epsilon^{7/2}\right)$ scaling of the corrected alg2 smoothing factor and $D_{\text{err}}\sim O\left(\epsilon^{6}\right)$ scaling of the tanh and erf smoothing factors are not the same as in the forward problem, which, as we recall, gives $u(x)\sim O\left(\epsilon^{2}\right)$ and $u(x)\sim O\left(\epsilon^{3}\right)$, respectively. Thus, our results highlight the need for further error analysis of MRS inverse problems.

We find that surface-oriented regularizations, which distribute force density in a way that is predominantly tangent to the boundary, also reduce error in the test problem of determining the drag on a sphere, especially when using smaller values of ϵ. The no-slip boundary condition on the sphere is also more effectively enforced away from the discretization points. Applying near-field corrections to surface-oriented blobs may allow for further reduction of error. We leave a detailed error analysis of surface-oriented regularizations to future work.

Aside from accuracy, computational expense is also an important factor when choosing a regularization. There are two main sources of computational expense. The first is the time necessary to evaluate the regularized Stokeslet at the discretization points. For time-dependent problems, Stokeslet evaluations must be repeated at each time step. We therefore assess the computational expense of Stokeslet evaluations via the method described in Appendix B. For the radial regularizations, whose smoothing factors appear in Table 1, Table A1 gives the time to compute the functions $h_{1}$ and $h_{3}$, which change in (18) depending on the regularization used. Unsurprisingly, the alg2 regularization is the simplest and the fastest to evaluate. The algebraic regularizations (alg2 and alg4) are fastest, followed by the tanh and then erf regularizations. The corrected regularizations are more computationally expensive than their uncorrected counterparts, but only by a factor of two or less. Given our results for both forward and inverse problems, the (corrected) alg2-c, alg4-c, and tanh-c regularizations provide a good trade-off between accuracy and computational cost. The erf-c regularization achieves the best accuracy but is comparatively more expensive. Near-field error limits the accuracy of all uncorrected regularizations, so there is less benefit for the added computational cost of the alg4, tanh, and erf regularizations even though they have smaller far-field errors. The surface-oriented regularization $G_{\epsilon }^{a}$ takes about twice as long to compute as the radial alg2 regularization in our testing. This may seem surprising given its relatively lengthy formula, given in Appendix B by (A5). However, many common terms appear in (A8) and (A9) which do not need to be repeatedly calculated for each evaluation of $G_{\epsilon }^{a}$.

The second major source of computational expense is the time necessary to solve the linear system given by (35), which applies only to the inverse problem. For our test problem of a translating sphere with n=4096 points, allocation and assembly of the matrix, which requires $n^{2}$ Stokeslet evaluations, took approximately 5 s (25% of the computational time), while the solution of the linear system took approximately 15 s (75%) when using the (relatively expensive) erf-c regularization. Thus, the cost of Stokeslet evaluations is relatively small for inverse problems, and the added cost of using a more expensive but accurate regularization is likely well worth the improved accuracy.

The condition number of the linear system in (35) is also considered for the different regularizations. We report these condition numbers in Appendix C, Figure A1. The condition numbers that we observe for the uncorrected regularizations are similar to those reported previously in the literature [33,42]. The condition number increases with ϵ, indicating that interaction between different discretization points increases sensitivity to errors. For the radial regularizations, condition numbers for the corrected blobs are larger than those for the uncorrected blobs. Interestingly, for larger values of ϵ, the erf and erf-c regularizations have significantly larger condition numbers than those of the other regularization choices. The condition numbers of the surface-oriented regularizations are slightly larger than that of the radial alg2 regularization (Figure A1c), except at ϵ=0.1, the largest value considered, where it is significantly larger. In general, though, condition numbers remain unproblematic for values of ϵ where the error in the drag is minimized, indicated by the shaded regions in Figure A1.

Among the radial regularizations, our results suggest that the tanh-c regularization may be a good general choice, which maintains a good balance between low computational expense, low condition number, and high accuracy for both forward and inverse problems.

We note that regularized Brinkman flows have been derived at the level of a biharmonic potential function, analogous to B that appears in (11) [28,52,53]. This regularization was achieved by replacing singular terms containing factors of 1/r with terms containing factors of $1/\sqrt{r^{2}+\epsilon^{2}}$. Our method generalizes and formalizes this approach, and the same methodologies could be applied to Brinkman flows.

## Figures and Tables

**Table 1.** Radially symmetric smoothing factors $s_{1}$ in use in (17) and their corresponding blob functions $\phi_{1}$. Here, r=‖r‖=‖x−y‖ for a point force at y. The “label” corresponds to that used in figure captions for the particular regularized Stokeslets and blobs that correspond to these smoothing factors. Smoothing factors are given with ϵ=1; recall that $s_{\epsilon }=s_{1}(r/\epsilon )$. The correction is the term added to the original smoothing factor (in the second column) to satisfy (25) at discretization points on the boundary. Formulas for $h_{1}$ and $h_{3}$ from (18) are given separately in Appendix B, Table A1.

Label	$s_{1}$	Correction Term	$\phi_{1}$	Correction Term
alg2	$\frac{r}{\sqrt{r^{2}+1}}$	$\frac{r}{{\left(r^{2}+1\right)}^{3/2}}$	$\frac{15}{8\pi {\left(r^{2}+1\right)}^{7/2}}$	$-\frac{15\left(4r^{2}-3\right)}{8\pi {\left(r^{2}+1\right)}^{9/2}}$
alg4	$\frac{r\left(2r^{2}+3\right)}{2{\left(r^{2}+1\right)}^{3/2}}$	$\frac{3r}{{\left(r^{2}+1\right)}^{5/2}}$	$\frac{15\left(5-2r^{2}\right)}{16\pi {\left(r^{2}+1\right)}^{9/2}}$	$\frac{15\left(8r^{4}-40r^{2}+15\right)}{16\pi {\left(r^{2}+1\right)}^{11/2}}$
tanh	tanh(r)	$2\;\text{ln}\;2\;\text{tanh}\;r\;{\text{sech}}^{2}r$	$\frac{\left(r+4\;\text{tanh}\;r-3r\;{\text{tanh}}^{2}\;r\right){\text{sech}}^{2}r}{4\pi r}$	See (*) below.
Erf	erf(r)	$\frac{2re^{-r^{2}}}{\sqrt{\pi }}$	$\frac{\left(5-2r^{2}\right)e^{-r^{2}}}{2\pi^{3/2}}$	$\frac{\left(4r^{4}-20r^{2}+15\right)e^{-r^{2}}}{2\pi^{3/2}}$
$\frac{\text{ln}\;2}{\pi r}\left(-15r{\;\text{sech}}^{2}\;r\;{\text{tanh}}^{2}\;r+2r-12\;{\text{tanh}}^{3}\;r+8\;\text{tanh}\;r\right){\text{sech}}^{2}\;r$.				(*)

## Data Availability

Julia code and computational results are available on request.

## Appendix A. Moments of Radial Blob Functions

We would like to relate the radial smoothing factors discussed in Section 2.3 to the moments of the corresponding blob function. We assume that $s_{\epsilon }$ satisfies the properties given in Theorem 1. Due to the scaling relation in (7), we may let ϵ=1 without loss of generality. Since $\phi_{1}$ is radially symmetric, we may transform to spherical coordinates and write the n-th moment of $\phi_{1}$ as

(A1)
$${ℳ}_{n}\phi_{1}≔C_{n}\int_{0}^{\infty }\;r^{n+2}\phi_{1}(r)\text{d}r$$

for n≥0. Note that we simplify notation by writing ϕ as a function of r=‖r‖ rather than the vector r. Due to radial symmetry, we have that $C_{n}=0$ for odd n and only the even moments of $\phi_{\epsilon }$ are nonzero. Multiplying both sides of this equation by $r^{n+2}$, integrating by parts, and utilizing (19), we find that

$$8\pi \int_{0}^{\infty }\;r^{n+2}\phi_{1}\left(r\right)\text{d}r=-{\left.r^{n+2}s_{1}^{\prime \prime }\left(r\right)\right|}_{0}^{\infty }+\left(n-2\right)\left({\left.r^{n+1}s_{1}^{\prime }\left(r\right)\right|}_{0}^{\infty }-\left(n+1\right)\int_{0}^{\infty }\;\;r^{n}s_{1}^{\prime }\left(r\right)\text{d}r\right),$$

where a prime indicates differentiation with respect to r. For ${ℳ}_{n}\phi_{1}$ to be convergent, we require that

(A2)
$$1-s_{1}(r)=O\left(r^{-n-\alpha }\right)$$

for α>0 as r→∞. In this case, all terms being evaluated at the integration limits vanish and

$$8\pi \int_{0}^{\infty }\;r^{n+2}\phi_{1}\left(r\right)\text{d}r=-\left(n-2\right)\left(n+1\right)\int_{0}^{\infty }\;r^{n}s_{1}^{\prime }\left(r\right)\text{d}r.$$

Replacing $s_{1}^{\prime }$ with $-{\left(1-s_{1}\right)}^{\prime }$ in this equation, integrating by parts once more, and using (20b) and (A2), we obtain

(A3)
$$8\pi \int_{0}^{\infty }\;r^{n+2}\phi_{1}(r)\text{d}r=\left\{\begin{array}{ll} 2 & n=0\\ -n(n-2)(n+1)\int_{0}^{\infty }\;\;r^{n-1}\left[1-s_{1}(r)\right]\text{d}r & n\geq 1. \end{array}\right.$$

Equation (A3) combines with (A1) to yield

(A4)
$${ℳ}_{n}\phi_{1}=\left\{\begin{array}{ll} 1 & n=0\\ -\frac{1}{8\pi }C_{n}n(n-2)(n+1)\int_{0}^{\infty }\;\;r^{n-1}\left[1-s_{1}(r)\right]\text{d}r & n\geq 1, \end{array}\right.$$

where we have used the fact that $C_{0}=4\pi$. As expected, (A4) shows that ${ℳ}_{0}\phi_{1}=1$ for any appropriate smoothing factor that satisfies the properties in (20) and (22). It also shows that ${ℳ}_{2}\phi =0$ as long as (A2) with n=2 holds.

## Appendix B. Expressions and Computational Cost of Regularized Stokeslets

The regularized Stokeslets derived from the smoothing factors described in Section 2.3 and summarized by Table 1 require the evaluation of the functions $h_{1}$ and $h_{3}$ that appear in (18). These functions are given in Table A1. We also give the time taken to evaluate $h_{1}$ and $h_{3}$ relative to the alg2 regularization, which corresponds to the commonly used 7/2-blob given by (2). All of the functions were implemented in Julia v1.8 and time trials were conducted using BenchmarkTools.jl v1.3.1. Note that the times reported reflect computation of both $h_{1}$ and $h_{3}$ together in the same Julia function h1h3. A sample of 1000 evaluations of h1h3 for the alg2 regularization took 3.49 μs to complete on an AMD Ryzen^™^ 7 PRO 4750U CPU, which is used as the reference value in Table A1. All reported performance measurements use the minimum time out of 10,000 samples.

We also consider the explicit formula for $G_{\epsilon }^{a}$, given by (12) as

(A5)
$$G_{\epsilon }^{a}=\left(I\nabla^{2}-\nabla \nabla \right)B_{\epsilon }^{a}.$$

Recall that $B_{\epsilon }^{a}=B_{\epsilon }^{a}(\rho ,z)$, given by (31), is symmetric about the z-axis, where ρ,θ, and z define a cylindrical polar coordinate system. In this case, we may write

(A6)
$$\nabla \nabla B_{\epsilon }^{a}\left(\rho ,z\right)=\frac{\partial^{2}B_{\epsilon }^{a}}{\partial \rho^{2}}\overset{ˆ}{\rho }\overset{ˆ}{\rho }+\frac{1}{\rho }\frac{\partial B_{\epsilon }^{a}}{\partial \rho }\overset{ˆ}{\theta }\overset{ˆ}{\theta }+\frac{\partial^{2}B_{\epsilon }^{a}}{\partial \rho \partial z}\left(\overset{ˆ}{\rho }\overset{ˆ}{z}+\overset{ˆ}{z}\overset{ˆ}{\rho }\right)+\frac{\partial^{2}B_{\epsilon }^{a}}{\partial z^{2}}\overset{ˆ}{z}\overset{ˆ}{z},$$

where $\overset{ˆ}{\rho },\;\overset{ˆ}{\theta }$, and $\overset{ˆ}{z}$ are the unit vectors in the ρ−,θ-, and z-directions, respectively. Taking the trace of (A6) gives the Laplacian of $B_{\epsilon }^{a}$ as

(A7)
$$\nabla^{2}B_{\epsilon }^{a}=\frac{1}{\rho }\frac{\partial }{\partial \rho }\left(\rho \frac{\partial B_{\epsilon }^{a}}{\partial \rho }\right)+\frac{\partial^{2}B_{\epsilon }^{a}}{\partial z^{2}}.$$

The derivatives that appear in (A6) and (A7) are given by

(A8a)
$$\frac{\partial B_{\epsilon }^{a}}{\partial \rho }=\frac{\rho -\epsilon a}{\sqrt{\epsilon^{2}+z^{2}+(\rho -\epsilon a{)}^{2}}}+\frac{(\rho +\epsilon a{)}^{2}}{\sqrt{\epsilon^{2}+z^{2}+(\rho +\epsilon a{)}^{2}}}$$

(A8b)
$$\frac{\partial^{2}B_{\epsilon }^{a}}{\partial \rho^{2}}=\frac{\epsilon^{2}+z^{2}}{{\left(\epsilon^{2}+z^{2}+(\rho +\epsilon a{)}^{2}\right)}^{3/2}}+\frac{\epsilon^{2}+z^{2}}{{\left(\epsilon^{2}+z^{2}+(\rho -\epsilon a{)}^{2}\right)}^{3/2}}$$

(A8c)
$$\frac{\partial^{2}B_{\epsilon }^{a}}{\partial \rho \partial z}=\frac{z(\epsilon a-\rho )}{{\left(\epsilon^{2}+z^{2}+(\rho -\epsilon a{)}^{2}\right)}^{3/2}}-\frac{z(\rho +\epsilon a{)}^{2}}{{\left(\epsilon^{2}+z^{2}+(\rho +\epsilon a{)}^{2}\right)}^{3/2}}$$

(A8d)
$$\frac{\partial^{2}B_{\epsilon }^{a}}{\partial z^{2}}\;=\frac{\epsilon^{2}+(\rho -\epsilon a{)}^{2}}{{\left(\epsilon^{2}+z^{2}+(\rho -\epsilon a{)}^{2}\right)}^{3/2}}+\frac{\epsilon^{2}+(\rho +\epsilon a{)}^{2}}{{\left(\epsilon^{2}+z^{2}+(\rho +\epsilon a{)}^{2}\right)}^{3/2}}$$

and the Laplacian is given by

(A9)
$$\nabla^{2}B_{\epsilon }^{a}=\frac{\epsilon^{2}\rho +(2\rho +\epsilon a)\left(\epsilon^{2}+z^{2}+(\rho +\epsilon a{)}^{2}\right)}{\rho {\left(\epsilon^{2}+z^{2}+(\rho +\epsilon a{)}^{2}\right)}^{3/2}}+\frac{\epsilon^{2}\rho +(2\rho -\epsilon a)\left(\epsilon^{2}+z^{2}+(\rho -\epsilon a{)}^{2}\right)}{\rho {\left(\epsilon^{2}+z^{2}+(\rho -\epsilon a{)}^{2}\right)}^{3/2}}.$$

Substitution of (A6) and (A9) into (A5) gives an explicit expression for $B_{\epsilon }^{a}$.

**Table A1.** Functions $h_{1}$ and $h_{3}$ that are used in (18) to generate regularized Stokeslets by differentiating the radial smoothing factors given in Table 1. We have set ϵ=1 for simplicity, but note that these functions obey the scaling properties $h_{m}(\epsilon ;r)=\epsilon^{-m}h_{m}(1,r/\epsilon )$ for m=1,3. The last column gives the time needed to evaluate both $h_{1}$ and $h_{3}$ together, relative to the alg2 regularization.

Label	$h_{1}(1;r)$	$h_{3}(1;r)$	Time
alg2	$\frac{r^{2}+2}{{\left(r^{2}+1\right)}^{3/2}}$	$\frac{1}{{\left(r^{2}+1\right)}^{3/2}}$	1.00
alg4	$\frac{2r^{4}+5r^{2}+6}{2{\left(r^{2}+1\right)}^{5/2}}$	$\frac{2r^{2}+5}{2{\left(r^{2}+1\right)}^{5/2}}$	1.06
tanh	$\frac{{\text{tanh}}^{2}\;r}{r}+{\text{sech}}^{2}r$	$\frac{\text{tanh}\;r}{r^{2}}-\frac{{\text{sech}}^{2}r}{r^{3}}$	2.07
erf	$\frac{\text{erf}r}{r}+\frac{2e^{-r^{2}}}{\sqrt{\pi }}$	$\frac{\text{erf}r}{r^{3}}-\frac{2e^{-r^{2}}}{\sqrt{\pi }r^{2}}$	5.26
alg2-c	$\frac{r^{4}+2r^{2}+4}{{\left(r^{2}+1\right)}^{5/2}}$	$\frac{r^{2}+4}{{\left(r^{2}+1\right)}^{5/2}}$	1.06
alg4-c	$\frac{2r^{6}+7r^{4}+2r^{2}+12}{2{\left(r^{2}+1\right)}^{7/2}}$	$\frac{2r^{4}+7r^{2}+20}{2{\left(r^{2}+1\right)}^{7/2}}$	2.04
tanh-c	$\left(2\;\text{ln}\;2{\;\text{sech}}^{2}r+1\right)\frac{\text{tanh}\;r}{r}+\left(6\;\text{ln}\;2{\;\text{sech}}^{2}r-4\;\text{ln}\;2+1\right){\;\text{sech}}^{2}r$	$\left(2\;\text{ln}\;2{\;\text{sech}}^{2}r+1\right)\frac{\text{tanh}\;r}{r^{3}}-\left(6\;\text{ln}\;2{\;\text{sech}}^{2}r-4\;\text{ln}\;2+1\right)\frac{{\;\text{sech}}^{2}r}{r^{2}}$	2.56
erf-c	$\frac{\text{erf}r}{r}-\frac{\left(4r^{2}-6\right)e^{-r^{2}}}{\sqrt{\pi }}$	$\frac{\text{erf}r}{r^{3}}+\frac{\left(4r^{2}-2\right)e^{-r^{2}}}{\sqrt{\pi }r^{2}}$	5.31

The blob function, $\phi_{\epsilon }^{a}=-\nabla^{4}B_{\epsilon }^{a}$, is given by taking the Laplacian of (A9). The resulting expression is quite lengthy but straightforward to obtain using, e.g., a computer algebra system. We therefore do not render it here.

## Appendix C. Matrix Condition Numbers

Skeel’s condition number of the matrices resulting from the linear system given by (35) are reported in Figure A1. Skeel’s condition number is defined for a matrix M as

$$\kappa_{S}(M)={\left\||M|\left|M^{-1}\right|\right\|}_{\infty }$$

where |⋅| denotes the componentwise absolute value, $|M{|}_{ij}=\left|M_{ij}\right|$, and $\|\cdot {\|}_{p}$ denotes the matrix p-norm. Generally, $\kappa_{S}(M)$ is of the same order of magnitude as the usual condition number, defined by $\kappa (M)=\|M{\|}_{2}{\left\|M^{-1}\right\|}_{2}$, but has the advantage of being faster to compute for large matrices. For example, using the erf-c blob with n=4096 discretization points and $\epsilon =0.07,\;\kappa =3.68\times 10^{7}$ and $\kappa_{S}=2.73\times 10^{7}$.

## Abbreviations

The following abbreviations are used in this manuscript:

MRS: Method of Regularized Stokeslets
IB: Immersed Boundary
